# Transcranial iTBS Combined With Trans‐Spinal iTBS Targeting PDE1A/cAMP/PKA Axis Regulates Neural Regeneration After Spinal Cord Injury

**DOI:** 10.1111/cns.70525

**Published:** 2025-07-23

**Authors:** Yingxue Fu, Xianbin Wang, Xingyu Chen, Shuang Wu

**Affiliations:** ^1^ Department of Rehabilitation Medicine The Affiliated Hospital of Guizhou Medical University Guiyang Guizhou China; ^2^ Department of Clinical Medicine Guizhou Medical University Guian New District Guizhou China

**Keywords:** cAMP, iTBS, neural regeneration, PDE1A, PRKACA, spinal cord injury, synaptic plasticity

## Abstract

**Aims:**

To explore the impact of intermittent theta burst stimulation (iTBS) treatment at various targets on spinal cord injury (SCI), as well as the effects of dual‐target iTBS therapy on neurological functional recovery in rats with SCI and its underlying mechanisms.

**Methods:**

Using an improved Allen's method, an incomplete C6 SCI model was established. Postoperatively, the rats with SCI underwent transcranial iTBS, trans‐spinal iTBS, or dual‐target iTBS. Neurological functional recovery and synaptic function following SCI were evaluated through behavioral tests, footprint analysis, electrophysiological assessments, pathological staining, transmission electron microscopy, Golgi staining, and immunofluorescence staining. The expression of relevant proteins and genes was assessed using Western blotting and qRT‐PCR. Proteomic and metabolomic sequencing analyses of spinal cord tissue from each group were conducted to investigate specific mechanisms. Additionally, lentivirus was used to infect primary neurons to elucidate the effect of PDE1A. Furthermore, lentivirus was applied to SCI rats to explore the influence of PDE1A on neurological and synaptic functions following SCI.

**Results:**

Compared with the single‐target iTBS group, dual‐target iTBS treatment significantly improved motor function and reduced the damaged area of the spinal cord in SCI rats. Following dual‐target iTBS intervention, SCI rats exhibited improvements in neural function and synaptic function. Sequencing analysis identified the protein PDE1A present in all groups, and the protein interaction network revealed that PDE1A is involved in the cAMP signaling pathway, with an increase in PDE1A expression observed after SCI. Additionally, inhibiting PDE1A promoted the expression of cAMP and protein kinase cAMP‐activated catalytic subunit alpha (PRKACA) in primary neurons, thereby facilitating synapse function in primary neurons. Inhibition of PDE1A also improved neural connection and synaptic reconstruction in SCI rats.

**Conclusion:**

Compared with single‐target iTBS treatment, dual‐target iTBS treatment promotes the recovery of motor function and spinal cord tissue repair more effectively in SCI rats. Dual‐target iTBS may promote neural regeneration and synaptic remodeling after SCI by regulating the PDE1A‐cAMP‐PKA signaling pathway, thereby improving neurological dysfunction.

## Graphical abstract

Dual‐target iTBS promotes neural regeneration and synaptic reconstruction after SCI via the PDE1A/cAMP/PKA signaling pathway. Inhibiting PDE1A promotes neural growth and synaptic function.

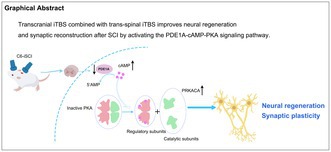

## 1 Introduction

Spinal cord injury (SCI) interrupts the neural connections between the upper central nervous system (CNS) and the spinal cord, leading to motor, sensory, and autonomic dysfunctions below the level of the SCI, often resulting in severe disabilities [1]. In spinal cord injuries, cervical SCI is the most common, accounting for 46.02% [2]. The persistence of functional impairment is mainly attributed to the limited capacity of neural regeneration and the reconstruction of functional connections in injured spinal cord neurons [3]. The pathological processes following SCI are generally divided into primary and secondary injury. Primary injury includes events such as vascular rupture and disruption of spinal cord nerve fiber bundles, whereas secondary injury encompasses neuroinflammation, demyelination, and astrocyte proliferation [4, 5]. These events exacerbate spinal cord tissue damage and inhibit axonal regeneration [6]. Therefore, investigating the mechanisms underlying neural regeneration after SCI has the potential to provide clinical benefit.

Transcranial magnetic stimulation (TMS) alters the membrane potential of nerve cells, affecting the excitability of nervous tissue and neural electrical activity, thereby triggering a series of physiological and biochemical responses [7, 8]. It was reported that high‐frequency TMS facilitated the transmission of electrical signals from the brain to the spinal cord and promoted the recovery of neural regeneration and conduction functions [9, 10]. Moreover, applying TMS at the injured spinal cord promoted the expression of GAP43 and 5‐HT and facilitated axonal regeneration [11]. The treatment goal after SCI is to find safe and reliable methods to promote the repair and reactivation of remaining neural connections. Research indicates that a single transcranial cortical target is insufficient for neural circuit reconstruction, necessitating a more comprehensive approach with multiple targets and multimodal interventions to rebuild sensorimotor circuits [12]. As a pattern of TMS, intermittent theta burst stimulation (iTBS) induces long‐term potentiation, alters neural plasticity, and achieves lasting changes in cortical excitability with fewer pulses and a shorter duration [13, 14, 15, 16, 17]. Consequently, we utilized combined transcranial iTBS with trans‐spinal iTBS to treat SCI. Our preliminary study has demonstrated that this approach improved motor function in patients with incomplete SCI [18]. However, further exploration of its potential mechanisms is needed.

Phosphodiesterases (PDEs) are enzymes that hydrolyze intracellular second messenger cyclic adenosine monophosphate (cAMP) or cyclic guanosine monophosphate (cGMP) [19, 20]. PDE hydrolyzes cAMP, and the intracellular level of cAMP is maintained by a delicate balance between the synthesis of adenylate cyclase and the degradation by PDE [21]. Protein kinase A (PKA), which is a cAMP‐dependent protein kinase, is an important downstream target of cAMP [22]. Studies have confirmed that the cAMP‐PKA signaling axis plays a vital role in the development of various neurological systems [23, 24, 25]. Research has reported that the autophagy protein ATG5 regulates neuronal excitability by modulating the cAMP/PKA signaling pathway at the synapse [26]. Increasing evidence suggests that inhibiting cAMP‐specific PDEs is an important strategy for coordinating neuroinflammation and regeneration in the CNS [27, 28, 29].

The purpose of this study was to compare the effects of single‐target iTBS and dual‐target iTBS on SCI in rats, investigate the efficacy of transcranial iTBS combined with trans‐spinal iTBS on neurological function in rats with cervical contusive SCI, and its potential mechanisms.

## 2 Materials and Methods

### 2.1 Animals

Eight‐week‐old SPF‐grade female Sprague Dawley (SD) rats weighing between 220 ± 20 g were purchased from Beijing Huafukang Biotechnology Co. Ltd. (Beijing, China, license no. SCXK (Jing) 2019‐0008). The animals were housed at a temperature of 22°C–26°C with a 12‐h light/dark cycle.

To compare the effects of single‐target iTBS and dual‐target iTBS on SCI, rats were randomly divided into five groups: (1) the sham group, which underwent sham surgery (*n* = 12); (2) the SCI group (*n* = 12), which underwent SCI operation; (3) the SCI + trans‐spinal iTBS group (*n* = 12), which underwent SCI surgery and trans‐spinal iTBS treatment after SCI; (4) the SCI + transcranial iTBS group (*n* = 12), which underwent SCI surgery and transcranial iTBS treatment after SCI; and (5) the SCI + dual‐target iTBS group (*n* = 12), which underwent SCI surgery and trans‐spinal iTBS followed by transcranial iTBS treatment after SCI.

To explore the effects of dual‐target iTBS on neural function and synaptic remodeling in rats with SCI, as well as its specific mechanisms, rats were randomly divided into: sham surgery group (Sham group, *n* = 24), SCI group (*n* = 24), and SCI + dual‐target iTBS group (SCI + iTBS group, *n* = 24).

To investigate the effects of inhibiting PDE1A on rats with SCI, rats were randomly divided into: SCI + PDE1A negative control lentivirus group (SCI + NC group, *n* = 21) and SCI + PDE1A knockdown group (SCI + PDE1A KD group, *n* = 21). Every attempt was made to reduce the number of animals employed in the project and to ease the pain and suffering of the animals. All protocols related to animal experiments were approved by the Animal Experiment Ethics Committee of Guizhou Medical University (approval no. 2201537; approval date: September 2, 2022).

### 2.2 SCI Model Establishment and Treatment

A standard SCI model was established using a modification of Allen's method [30]. The rats were anesthetized with 1% pentobarbital sodium (50 mg/kg) by intraperitoneal injection, and the skin was prepared for the operation. A midline incision of approximately 1.5 cm on the back of the neck was determined under the guidance of bone markers. C6 vertebral laminae were fully exposed after blunt dissection of the paraspinal muscles. The spinous process and lamina were removed with microscissors to fully expose the spinal cord. We used the improved impactor device (Meyue Biotechnology Co. Ltd., Changsha, China) to prepare the SCI model in rats. After the hammer was accurately aligned to a specific spinal segment, the impact force caused by free fall from a mass of 10 g and a height of 25 mm was used to induce severe hematoma at the hit site [31]. Hemostatic sponge was applied for local hemostasis, and the separated muscles and skin were sutured layer by layer. The Sham group underwent only laminectomy, and the spinal cord remained intact. During the recovery from anesthesia, the rats were placed on a heating pad until fully awake. Postoperatively, penicillin was administered intraperitoneally for three consecutive days, and manual bladder expression was performed twice daily until spontaneous voiding returned.

To minimize the effects of handling, restraint, and stimulation, the rats were acclimatized to all experimental conditions, including the noise from magnetic stimulation and the homemade restraint device, 1 week prior to the iTBS intervention. We performed transcranial iTBS in the cerebral cortex region, trans‐spinal iTBS in the C6 spinal segment, or dual‐target iTBS using a magnetic stimulator (CCY‐II; Yiruide Medical Equipment New Technology Co. Ltd., Wuhan, China) connected to a round coil (diameter 6.4 cm) 24 h after SCI. Treatment was administered once daily for a total of 28 days. The Sham group and SCI group rats did not receive any treatment. Determination of resting motor threshold (RMT): In the minimum stimulus with at least five waves of motor evoked potential (MEP) exceeding 200 μV in 10 RMT tests, the stimulus intensity set in this study was 100% RMT, which is 26% of the maximum output intensity of the machine. The stimuli parameters set in this study consisted of 10 pulse bursts (each burst consisting of 3 pulses at 50 Hz) repeated with an interval of 200 ms (5‐Hz theta frequency) to form a sequence of 2 s of stimulation, followed by an 8‐s rest. This sequence was repeated 20 times, resulting in a total of 600 pulse stimulations.

**FIGURE 1:**
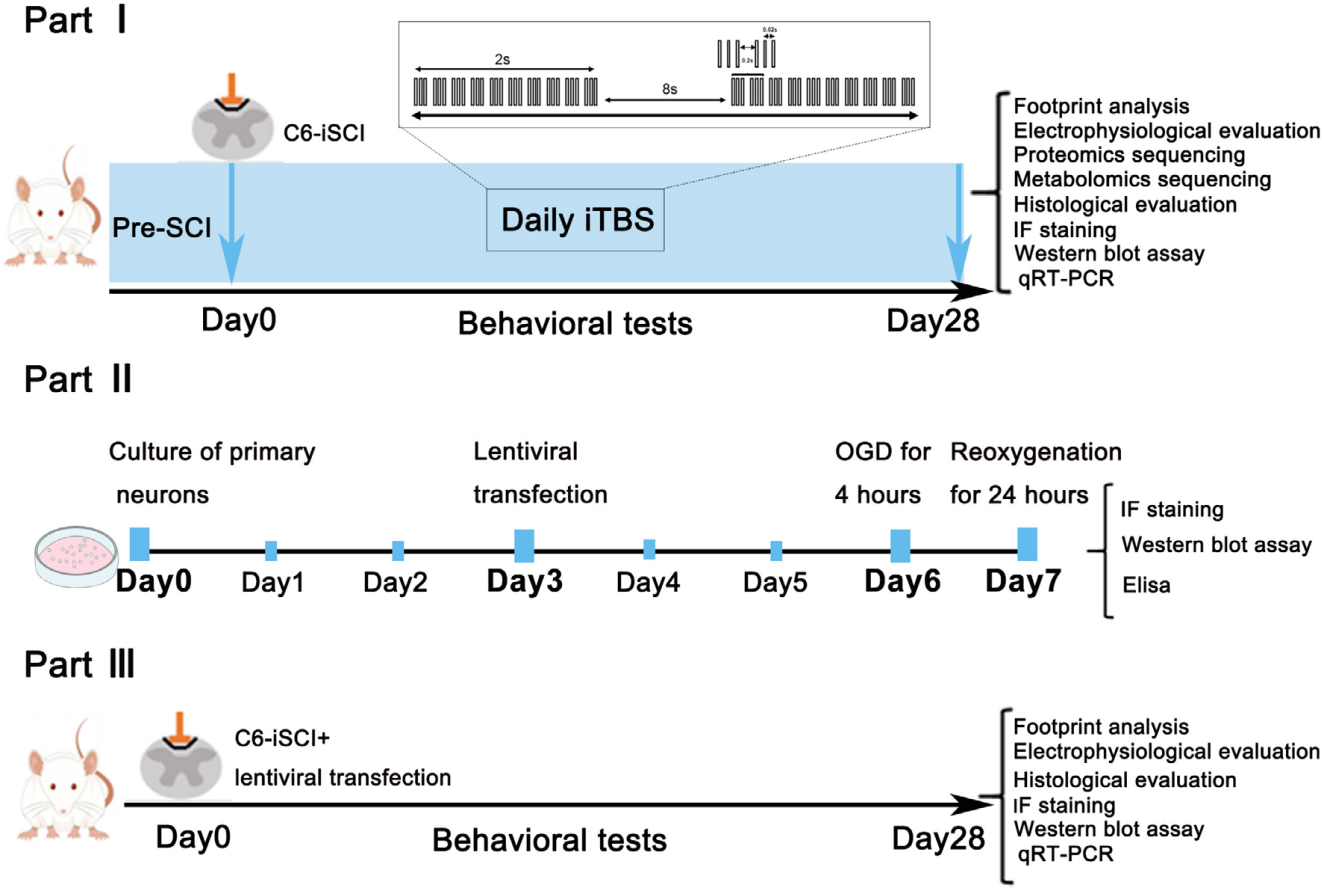
Experimental flowchart.

The lentivirus used for PDE1A knockdown was constructed by Shanghai Jikai Gene Technology Co. Ltd. (Shanghai, China). After successfully establishing the SCI model, a microinjection syringe was used to slowly inject the lentivirus (10 μL, lentiviral vector concentration of 2 × 10^7^ TU/mL) into the spinal cord around the injury site [32]. The needle was inserted vertically along the long axis of the spinal cord to a depth of 1.5 mm. After the injection, the needle was left in place for about 3 min before being slowly withdrawn to prevent the loss of the lentiviral suspension [33]. A flowchart of the experimental procedures is shown in Figure 1.

### 2.3 Behavioral Tests

The grid walking test was performed. A grid (metal grid measuring 1.5 m × 1.5 m, each square measuring 5 cm × 5 cm and a height of 50 cm) was used for testing. The rats were placed on the grid and allowed to walk freely. One observer counted the number of steps taken by the rats, while another observer recorded the number of times the hind limbs dropped within 30 steps. One step was defined as each hind limb (left and right) making contact with the grid once. It was recorded as a foot slip if the hind paw descended below the grid.

The ladder dexterity test was used to evaluate motor coordination of rats. The device, constructed from metal bars with a diameter of 3 mm and measuring 100 cm in length and 19 cm in height, was positioned 30 cm above the ground. Rat housing cages were situated at both ends of the device. The distance between the two side panels was adjusted to exceed the width of the rat by more than 1 cm to prevent the rats from turning. Additionally, the spacing between the rungs was randomly altered each day, varying between 1 and 3 cm, to mitigate the rats' ability to learn and adapt to the rung spacing. The average percentage of errors was recorded (number of steps missed, slipped, tripped, and fallen/total number of steps × 100%).

The hanging wire test was used to evaluate forelimb grip strength and motor coordination. A metal wire was stretched and suspended 60 cm above the ground, with a soft blanket positioned beneath to prevent injury to the rats. The rats were guided to grasp the metal wire with their forepaws, after which they were supported to hang independently. The scoring for the hanging wire test was based on a 5‐point scale: 0, immediately fall off; 1, have two front paws available to hang on the wire; 2, have two front paws hanging on the wire and trying to climb upward; 3, based on 2, add using one or two hind paws to grasp the wire to climb upward; 4, have four paws grasping the wire and wrapping the tail around the wire to stabilize the trunk. Each rat was tested three times with an interval of 5 min each, and the highest score in the three tests was selected for statistical analysis.

The rotarod test was used to evaluate the motor coordination and balance ability of rats. During the test, the forepaws and hind paws of the rats were gently positioned on the rotating rod, facing away from the direction of the rod's rotation. The rotation speed of the rod was gradually increased throughout the test, and the time until the rats fell off the rod was recorded.

For the footprint analysis, the forepaws and hind paws of the rats were painted with red and blue dyes, respectively. Rats were then allowed to walk in a straight path lined with white paper.

### 2.4 Electrophysiological Assessments

MEP and somatosensory evoked potential (SEP) were applied to evaluate neurological connectivity 28 days after SCI. After anesthetization, rats were fixed in a prone position. The MEP signals during stimulation were collected. Stimulating electrodes were placed on the skull surface, whereas ground electrodes were placed on the back to reduce interference. Receiving electrodes were inserted into the hind limb muscles. During the SEP test, the stimulating electrode was placed on the hind limb muscles, whereas the receiving electrode was positioned on the scalp.

### 2.5 Culture of Primary Cortical Neurons

The isolation and primary culture of cortical neurons were performed by the described protocols [34, 35]. Primary cultured cortical neurons were obtained from the cerebral cortex of 1‐day‐old SD rat pups. The brains were removed and placed in a dish containing ice‐cold high‐glucose DMEM (Gibco, New York, NY, USA), and the pia mater was removed for later use. Using tweezers, 2.0–3.0 mm thick sections of cortical tissue were placed in the dish and digested with papain (Solarbio, Beijing, China) for 20 min. After digestion, the tissue was gently triturated, and the resulting cell suspension was collected, filtered, centrifuged, and resuspended. The cells were then plated onto six‐well plates coated with poly‐L‐lysine (Sigma, USA) and incubated in a cell culture incubator at 37°C with 5% CO_2_. The neuron growth medium contained a neurobasal‐A medium (Gibco), with 2% B27 (Gibco), 1% penicillin–streptomycin (Gibco), and 1% L‐Glutamine (Solarbio). The culture medium was fully replaced on the first day, and subsequently, half of the medium was replaced every 2–3 days.

The lentivirus was dissolved at 4°C and gently put into the appropriate culture dish on the third day of primary neuron culture. An inverted fluorescence microscope (Olympus, Tokyo, Japan) was used to view the expression intensity of green fluorescence in the cells 72 h after the infection.

The oxygen–glucose deprivation/reoxygenation (OGD/R) model was conducted as previously described [34, 36]. The neuronal culture medium was replaced with glucose‐free DMEM, and the neurons were then placed in a 37°C incubator with an atmosphere of 95% N_2_ and 5% CO_2_ for a duration of 4 h. After 4 h, the glucose‐free medium was replaced with neuron growth medium, and the cells were cultured under aerobic conditions for 24 h.

### 2.6 Hematoxylin–Eosin Staining (H&E Staining)

The rats were anesthetized, and the left ventricle was used to carry out continuous perfusion using saline and 4% paraformaldehyde. The spinal cord tissue from the C6 spinal cord segment and adjacent segments was harvested. After gradient alcohol dehydration, the tissues were embedded in paraffin, sequentially sectioned (4 μm thick), and subjected to H&E staining as per manufacturer instructions (Solarbio).

### 2.7 Nissl Staining

Sections of the tissue, each 4 μm thick, were stained for 10 min with tartrazine purple staining solution (Solarbio), washed with deionized water, and then differentiated for a short while with differentiation solution.

### 2.8 Luxol Fast Blue Staining (LFB Staining)

Tissue sections (4 μm thick) were stained for 2 h at 65°C using Luxol Fast Blue solution (Solarbio). After using 95% ethanol to remove any remaining staining solution from the section, differentiation solution was used to distinguish the slice for 15 s, then 70% ethanol was used until the gray and white matter were well defined.

### 2.9 Immunofluorescence Staining (IF Staining)

Tissue sections (4 μm thick) were subjected to antigen retrieval with EDTA buffer (Proteintech, Wuhan, China). Cells were washed three times with phosphate buffer saline (PBS) and then fixed with 4% formaldehyde at room temperature for 30 min. Thereafter, they were permeabilized with 0.3% TritonX‐100 (Solarbio) in PBS and blocked with 10% goat serum (Solarbio) at room temperature, followed by incubation overnight at 4°C with the primary antibodies: rabbit anti‐CaMKII (1:250, ab52476; Abcam), rabbit anti‐MAP2 (1:200, 17490‐1‐AP; Proteintech), rabbit anti‐NF‐L (1:50, ab223343; Abcam), rabbit anti‐NF‐H (1:200, 18934‐1‐AP; Proteintech), mouse anti‐TUJ1 (1:200, M0805‐8; HUABIO), rabbit anti‐NeuN (1:300, ET1602‐12; HUABIO), rabbit anti‐PSD‐95 (1:200, 81106‐1‐RR; Proteintech), Synaptophysin (1:400, 67864‐1‐Ig; Proteintech). The next day, the slices and cells were incubated with the corresponding secondary antibodies (1:300, RGAR002/RGAR004/RGAM004; Proteintech) at room temperature for 1 h, and DAPI staining was used to identify the nuclei. Images were captured using a fluorescence microscope.

### 2.10 Western Blot Assay

Total proteins were extracted from the spinal cord tissue or primary neurons using RIPA lysis buffer (Solarbio). The protein concentration was detected using BCA kit (Solarbio). Next, the protein was denatured at high temperature. The denatured proteins were separated in sodium dodecyl sulfate–polyacrylamide gel electrophoresis. Then, the proteins were electrotransferred to a polyvinylidene fluoride membrane. After blocking with 5% skim milk for 1 h, the membranes were incubated at 4°C overnight with the following specific primary antibodies: mouse anti‐β‐actin (1:10,000, 66009‐1‐Ig; Proteintech), rabbit anti‐CaMKII (1:2000, ET1608‐47; HUABIO), rabbit anti‐PDE1A (1:1000, bs‐9967R; Bioss), mouse anti‐PRKACA (1:2000, 67491‐1‐Ig; Proteintech), rabbit anti‐PSD‐95 (1:2000, ET1602‐20; HUABIO), mouse anti‐Synaptophysin (1:50,000, 67864‐1‐Ig; Proteintech). The next day, the membranes were washed with tris buffered saline with tween (TBST) three times, and incubated with corresponding secondary antibodies (1:5000, SA00001‐1/SA00001‐2; Proteintech) at room temperature for 1 h. The bands were detected using enhanced chemiluminescence.

### 2.11 Enzyme‐Linked Immunosorbent Assay (ELISA)

The ELISA kit (Elabscience, Wuhan, China) was used to measure the levels of cAMP in spinal cord tissue and primary neurons. The absorbance at 450 nm was read on the microplate reader.

### 2.12 Quantitative Real‐Time PCR

Total RNA from spinal cord was extracted using TRIzol reagent (Invitrogen, Carlsbad, CA, USA). The first‐strand cDNA was synthesized using an RT kit (Yisheng, Shanghai, China). The cycle threshold (CT) was detected using an RT‐PCR kit (Yisheng), and the relative expression level of the target gene was calculated using the $2^{-\Delta \Delta C_{T}}$ method. The primer sequences are shown in Table 1.

**TABLE 1:** Primers for qRT‐PCR.

Gene name	Forward primer (5′–3′)	Reverse primer (5′–3′)
PDE1A	TTCCATGTTGCTGACGCTCT	AAGCTCTTCAGGTCCACAGC
PRKACA	CTCCTTTGGGCGAGTGAT	CTTGGCAAAACCGAAGTCT
β‐Actin	TGTCACCAACTGGGACGATA	GGGGTGTTGAAGGTCTCAAA

### 2.13 Transmission Electron Microscopy (TEM)

The spinal cord tissue was quickly placed in an electron microscope fixation solution and stored at 4°C for fixation. After washing three times with PBS, the tissue was fixed in a dark environment with 1% osmium tetroxide at room temperature for 2 h. The tissue was then dehydrated in ascending grades of alcohol before embedding the samples. After sectioning, the specimens were stained with a saturated solution of 2% uranyl acetate in alcohol and a 2.6% lead citrate solution. Finally, observation was conducted under a transmission electron microscope.

### 2.14 Golgi Staining

After rinsing the spinal cord tissue with deionized water to remove blood, the tissue was directly placed in Golgi staining fixative. It was then kept in a cool, ventilated area at 26°C in the dark for 14 days. After that, the processing solution was changed and treated for 1 h, followed by another change of processing solution and treatment at 4°C in the dark for 3 days. The samples were cut into 60 μm thick sections and then underwent imaging and scanning.

### 2.15 Sholl Analysis

The lengths of all dendrites on a neuron in several 400× magnification images from each section were measured to obtain the average dendrite length. Then, using ImageJ analysis software, a structural diagram of the neuronal cell body at the center of each 400× image was created. The Sholl analysis plugin was used to draw 10 concentric circles with a spacing of 10 μm centered around the cell body. The number of intersection points between the dendrites and the concentric circles was counted, and the sum of the intersection points for the 10 circles was calculated.

### 2.16 Statistical Analysis

Statistical analyses were performed using Prism 8.0.1 (GraphPad Software Inc., San Diego, CA, USA). The normality of the distribution of continuous variables was assessed using the Shapiro–Wilk normality test. If data are normally distributed with homogeneity of variance, two‐way analysis of variance (ANOVA) was used for comparing two or more groups across multiple time points followed by Bonferroni post hoc test; one‐way ANOVA was employed followed by Tukey's post hoc test for comparing multiple groups; unpaired *t*‐test was applied for comparing two groups. If data are normally distributed but exhibit heterogeneity of variance, Welch's ANOVA was used for multiple group comparisons followed by Games‐Howell's post hoc test; Welch's *t*‐test was employed for comparisons between two groups. If data do not follow a normal distribution, Kruskal–Wallis *H* test followed by Dunn's post hoc test was employed for the comparison of multiple groups. Statistical significance was set at *p* < 0.05.

## 3 Results

### 3.1 Dual‐Target iTBS Accelerates Functional Recovery and Spinal Cord Tissue Repair After SCI

**FIGURE 2:**
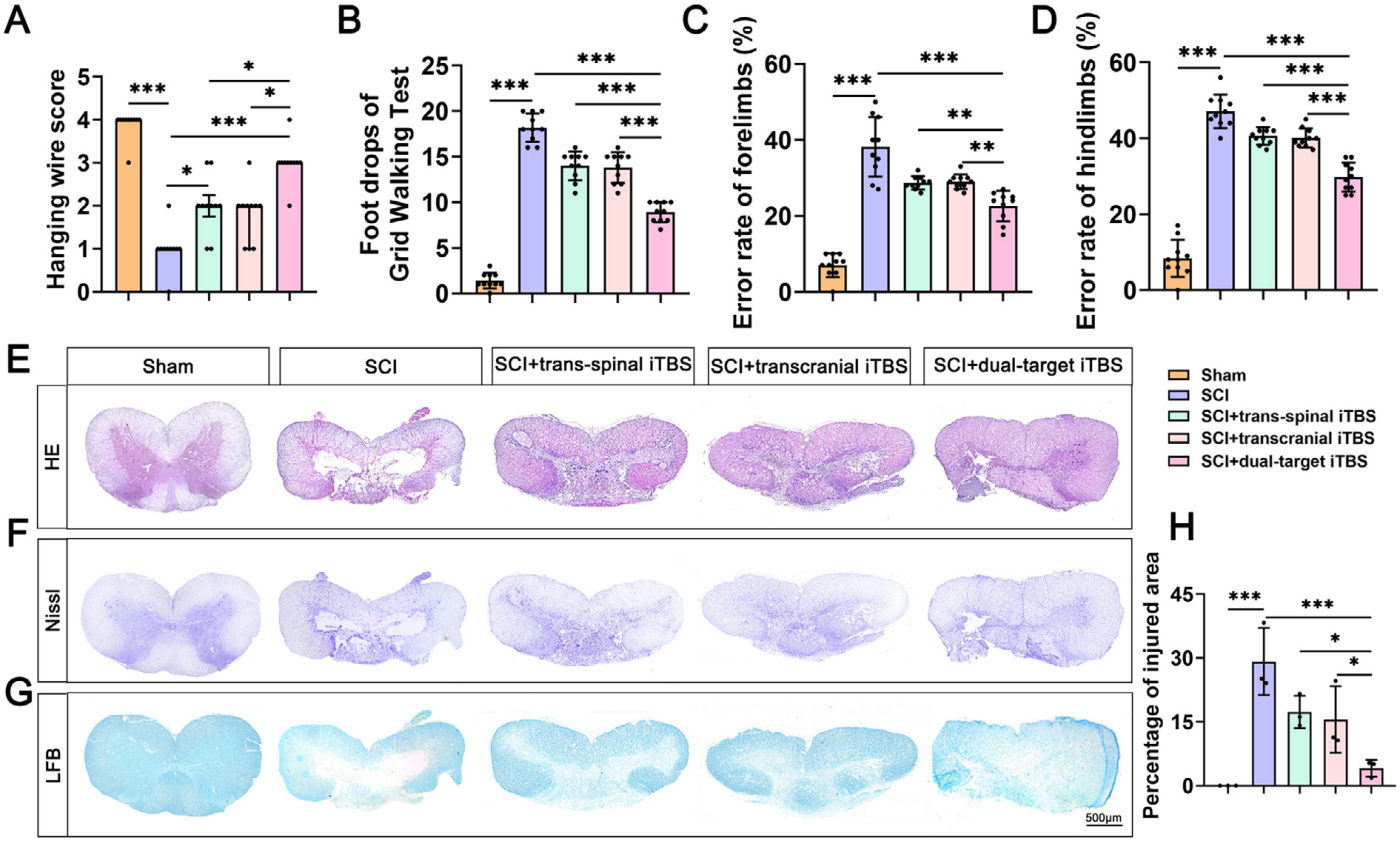
Dual‐target iTBS accelerates functional recovery and spinal cord tissue repair after SCI. (A) The hanging wire test at 28 days post‐operation (*n* = 10 per group). (B) The grid walking test 28 days post‐operation (*n* = 10 per group). (C) The error rates of forelimbs in the ladder dexterity test 28 days post‐operation (*n* = 10 per group). (D) The error rates of hindlimbs in the ladder dexterity test 28 days post‐operation (*n* = 10 per group). (E) Representative images of H&E staining. (F) Representative images of Nissl staining. (G) Representative images of LFB staining. (H) Quantification of H&E staining in E (*n* = 3 per group). The data of the hanging wire test are presented as median (IQR), and the remaining data were all presented as means ± SD. *p < 0.05, **p < 0.01, ***p < 0.001 (Kruskal–Wallis *H* test followed by Dunn's post hoc test [A], one‐way ANOVA followed by Tukey's post hoc test [B–D, H]).

In order to compare the therapeutic efficacy of single‐target iTBS (trans‐spinal iTBS, transcranial iTBS) and dual‐target iTBS for treating SCI, SCI rats were subjected to trans‐spinal iTBS, transcranial iTBS, and dual‐target iTBS treatment respectively. As demonstrated in Figure 2A–D, compared with the single‐target iTBS, the dual‐target iTBS significantly improved the hanging wire test scores, reduced hindlimb drops in the grid walking test, and decreased error rates of forelimbs and hindlimbs in the ladder dexterity test in rats with SCI. These results suggest that dual‐target iTBS stimulation more effectively enhances motor function recovery in rats with SCI. Additionally, HE staining revealed that treatment with dual‐target iTBS effectively prevented pathological damage after SCI (Figure 2H). The results demonstrate that dual‐target iTBS more effectively improves neurological function and promotes spinal cord tissue repair after injury.

### 3.2 Dual‐Target iTBS Improves Neurological Function After SCI

**FIGURE 3:**
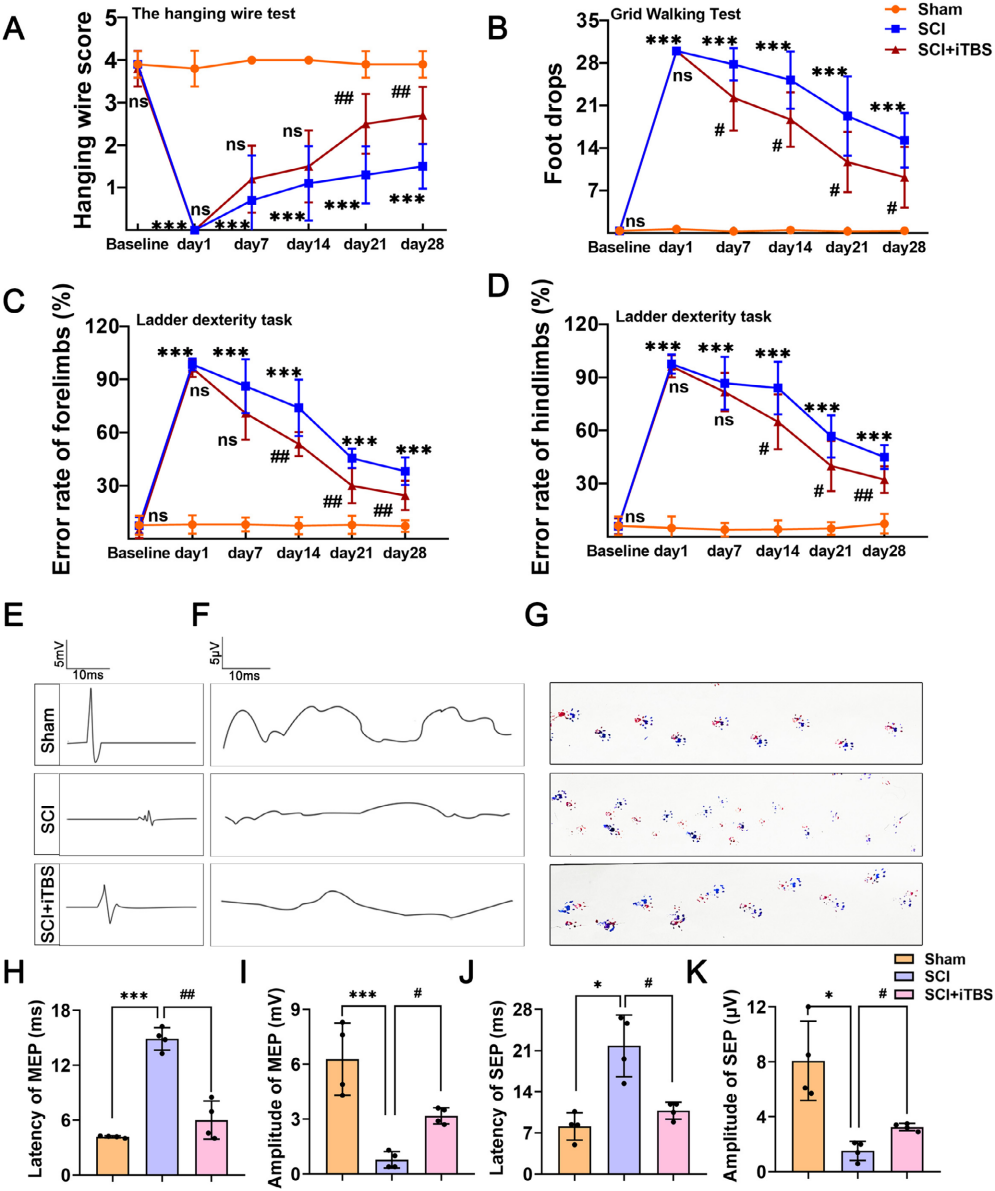
Dual‐target iTBS improves neurological function after SCI. (A) The hanging wire test over time (*n* = 10 per group). (B) The grid walking test over time (*n* = 10 per group). (C) The error rates of forelimbs in the ladder dexterity test over time (*n* = 10 per group). (D) The error rates of hindlimbs in the ladder dexterity test over time (*n* = 10 per group). (E) Representative images of MEP. (F) Representative images of SEP. (G) Representative images of footprint. (H) Quantification of MEP and latency period in E (*n* = 4 per group). (I) Quantification of MEP and amplitude period in E (*n* = 4 per group). (J) Quantification of SEP and latency period in F (*n* = 4 per group). (K) Quantification of SEP and amplitude period in F (*n* = 4 per group). The data are presented as means ± SD. No significance (ns) *p* > 0.05; compared to the Sham group, *p < 0.05, **p < 0.01, ***p < 0.001; compared to the SCI group, ^#^ *p* < 0.05, ^##^ *p* < 0.01, ^###^ *p* < 0.001 (two‐way ANOVA followed by Bonferroni post hoc test [A–D], Welch's ANOVA test followed by Games‐Howell's post hoc test [H–K]).

To investigate the effects of dual‐target iTBS on neurofunctional recovery after SCI, we assessed the motor function of rats using the hanging wire test, the ladder dexterity test, the grid walking test, and footprint analysis. The results indicated that the number of missteps in the grid walking test significantly decreased in the rats after 7, 14, 21, and 28 days of dual‐target iTBS intervention, in comparison to the SCI group (Figure 3B). Dual‐target iTBS led to a reduction in error rates on the ladder dexterity test for both forelimbs and hindlimbs following the14, 21, and 28‐day interventions and a significant increase in scores on the hanging wire test following 21 and 28‐day interventions in SCI rats (Figure 3A,C,D). Additionally, the results of the footprint analysis demonstrated that the footprints of the rats exhibited greater orderliness and improved coordination following the dual‐target iTBS intervention compared to the SCI group (Figure 3G). These findings suggest that dual‐target iTBS significantly enhances motor function in rats with SCI.

To further evaluate neural connectivity, we measured the MEP and SEP in rats with SCI (Figure 3E,F). As illustrated in Figure 3H–K, the amplitudes of MEP and SEP in the SCI group were significantly reduced, whereas the latencies were significantly prolonged in comparison to the Sham group. Additionally, dual‐target iTBS significantly increased the amplitudes of both MEP and SEP and shortened the latencies in rats with SCI, indicating that dual‐target iTBS enhanced neural connectivity. These results demonstrate that dual‐target iTBS significantly improves neural function in rats with SCI.

### 3.3 Dual‐Target iTBS Promotes Neural Regeneration After SCI

**FIGURE 4:**
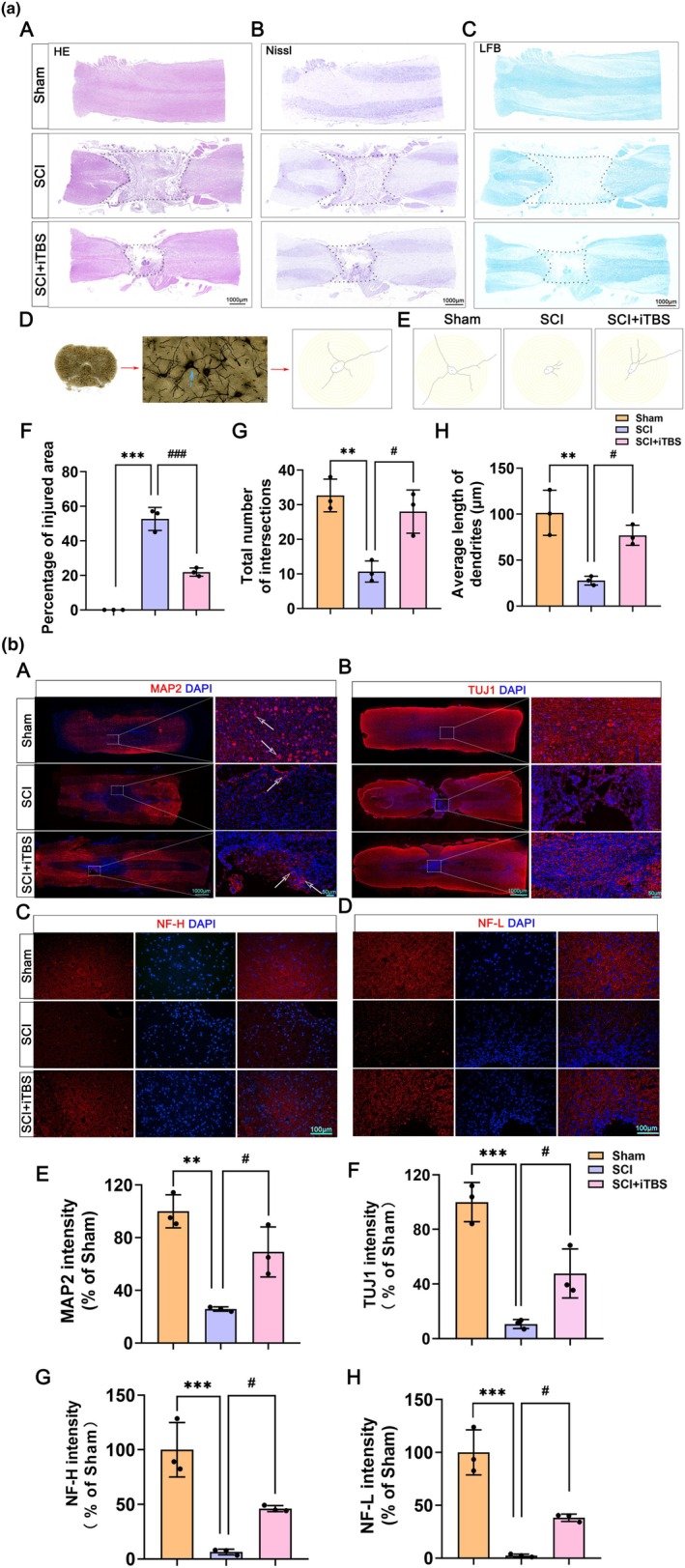
Dual‐target iTBS promotes neural regeneration after SCI. (a) (A) Representative images of H&E staining. (B) Representative images of Nissl staining. (C) Representative images of LFB staining. (D) Golgi staining and Sholl analysis schematic diagram. (E) Representative images of Golgi staining. (F) Quantification of H&E staining in A (*n* = 3 per group). (G) Total number of intersections between dendrites and concentric circles in E (*n* = 3 per group). (H) Average dendritic length in E (*n* = 3 per group). The data are presented as means ± SD. Compared to the Sham group, *p < 0.05, **p < 0.01, ***p < 0.001; compared to the SCI group, ^#^ *p* < 0.05, ^##^ *p* < 0.01, ^###^ *p* < 0.001; ns *p* > 0.05 (one‐way ANOVA followed by Tukey's post hoc test [F–H]). (b) (A) Representative images of IF staining of MAP2. (B) Representative images of IF staining of TUJ1. (C) Representative images of IF staining of NF‐H. (D) Representative images of IF staining of NF‐L. (E) Quantification of IF staining of MAP2 in A (*n* = 3 per group). (F) Quantification of IF staining of TUJ1 in B (*n* = 3 per group). (G) Quantification of IF staining of NF‐H in C (*n* = 3 per group). (H) Quantification of IF staining of NF‐L in D (*n* = 3 per group). The data are presented as means ± SD. Compared to the Sham group, *p < 0.05, **p < 0.01, ***p < 0.001; compared to the SCI group, ^#^ *p* < 0.05, ^##^ *p* < 0.01, ^###^ *p* < 0.001; ns *p* > 0.05 (one‐way ANOVA followed by Tukey's post hoc test [E–H]).

We investigated the effect of the dual‐target iTBS on the structural repair of injured spinal cord tissue. Compared with that in the injury group, a significant reduction in lesion volume was found in rats that received dual‐target iTBS treatment, indicating an improvement in neural pathway connectivity (Figure 4a[A–C,F]). Through Golgi staining and Sholl analysis, we further discovered that dual‐target iTBS significantly increased the average length of neuronal dendrites and the number of intersections between dendrites and concentric circles (Figure 4a[E,G–H]). Additionally, IF staining was conducted to assess the expression of microtubule‐associated protein 2 (MAP2), neuron‐specific class III β‐tubulin (TUJ1), neurofilament heavy chain (NF‐H), and neurofilament light chain (NF‐L) in spinal cord tissue. The findings revealed that, compared to the SCI group, the treatment group exhibited a significant increase in the expression of MAP2, TUJ1, NF‐H, and NF‐L at the site of SCI (Figure 4b[A–H]). These results suggest that dual‐target iTBS promotes neural regeneration following SCI.

### 3.4 Dual‐Target iTBS Promotes Synaptic Function After SCI

**FIGURE 5:**
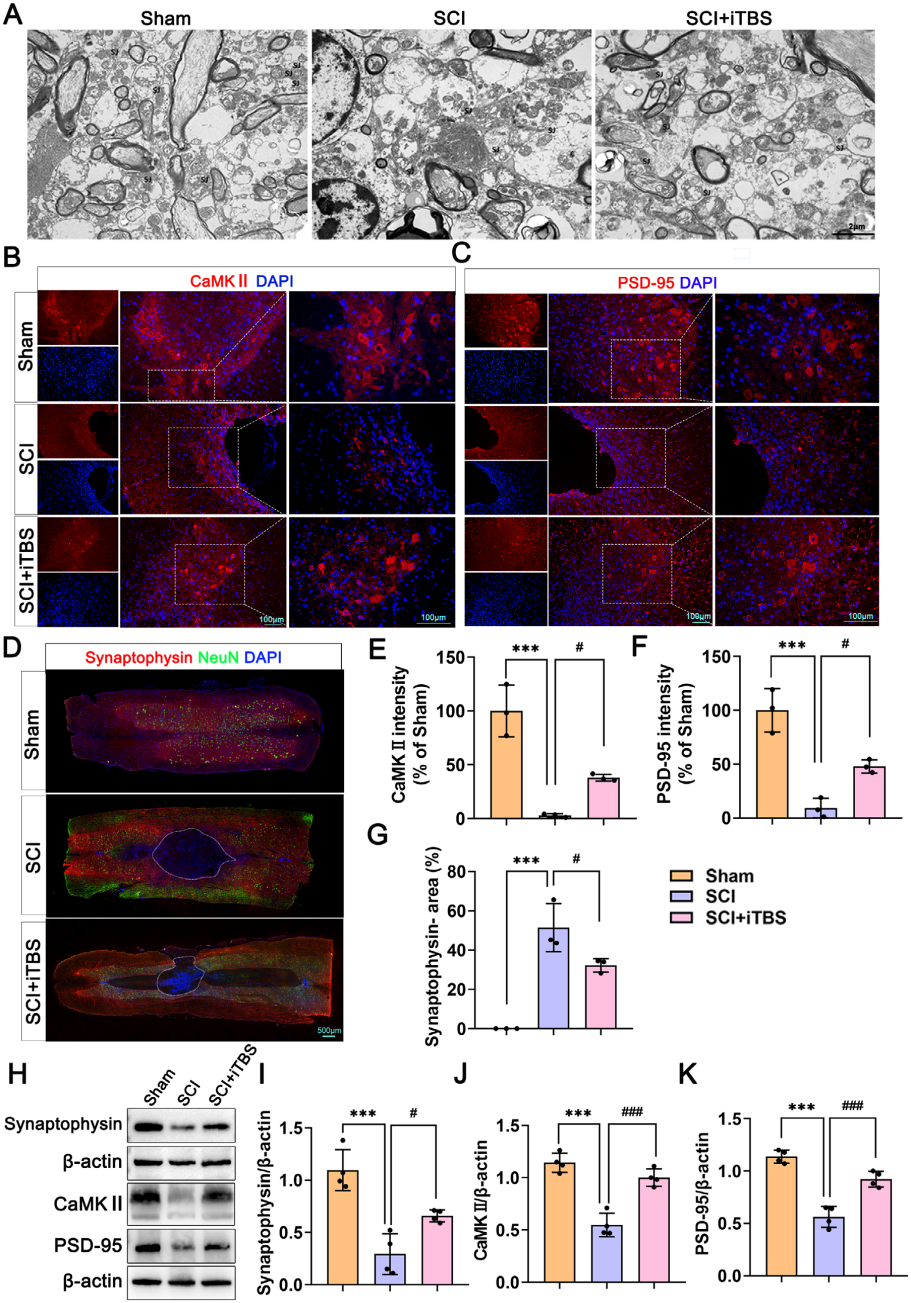
Dual‐target iTBS promotes synaptic function after SCI. (A) Representative images of TEM. (B–D) Representative images of IF staining of CaMKII, PSD‐95, and Synaptophysin/NeuN. (E–G) Quantification of IF staining of CaMKII, PSD‐95, and Synaptophysin‐negative area in B–D (*n* = 3 per group). (H) Western blots of Synaptophysin, PSD‐95, and CaMKII proteins in the Sham, SCI, and SCI + iTBS groups. (I–k) Quantification of Synaptophysin, PSD‐95, and CaMKII proteins expression levels in H (*n* = 4 per group). The data are presented as means ± SD. Compared to the Sham group, *p < 0.05, **p < 0.01, ***p < 0.001; compared to the SCI group, ^#^ *p* < 0.05, ^##^ *p* < 0.01, ^###^ *p* < 0.001; ns *p* > 0.05 (one‐way ANOVA followed by Tukey's post hoc test [E–G, I–K]).

To investigate the effect of dual‐target iTBS on neuronal synapses following SCI, we conducted TEM, IF staining, and Western blot assay. The TEM results indicated that in the Sham group, the dendritic spine matrix appeared relatively uniform, and the synaptic clefts were clearly defined. The SCI group exhibited a reduction in the number of synapses within the neural fleece, accompanied by significant swelling of the synaptic bodies. The axon terminal (T) membrane showed extensive damage and considerable edema, while the dendritic spine membrane was compromised, resulting in blurred synaptic clefts. In the SCI + iTBS group, the number of synaptic junction (SJ) in the neural fleece increased, although some localized membrane blurriness was observed. There was partial damage to the axon T membrane, characterized by slightly thicker and more continuous dense regions. The synaptic clefts were somewhat blurred (Figure 5A). These findings suggest that dual‐target iTBS significantly enhances both the quantity and structure of synapses. The IF staining and Western blot assay results demonstrated that dual‐target iTBS significantly decreased the synaptophysin‐negative area and increased the expression of synapse‐related proteins, including Synaptophysin, postsynaptic density protein 95 (PSD‐95), and calcium/calmodulin‐dependent protein kinase II (CaMKII) (Figure 5B–K). Collectively, these results indicate that dual‐target iTBS promotes synaptic remodeling in neurons following SCI.

### 3.5 Dual‐Target iTBS Improves Neurological Function After SCI Through the PDE1A/cAMP/PKA Signaling Pathway

**FIGURE 6:**
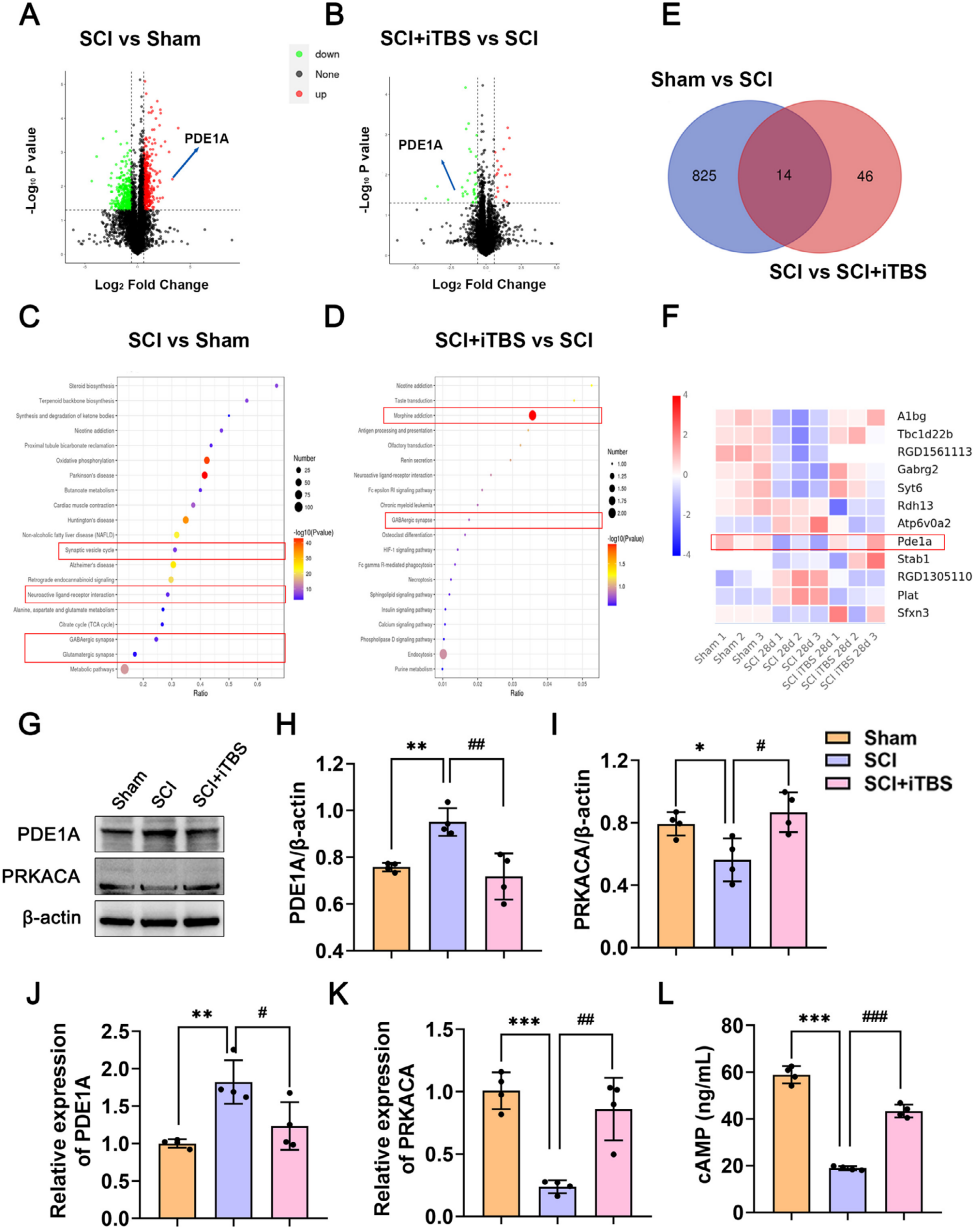
Dual‐target iTBS improves neurological function after SCI through the PDE1A/cAMP/PKA signaling pathway. (A, B) Volcano plot of upregulated and downregulated genes in the Sham, SCI, and SCI + iTBS groups. (C, D) KEGG enrichment analysis results. (E, F) The 14 proteins in the intersection of the Sham, SCI, and SCI + iTBS groups. (G) Western blots of PDE1A and PRKACA proteins in the Sham, SCI, and SCI + iTBS groups. (H, I) Quantification of PDE1A and PRKACA proteins expression levels in G (*n* = 4 per group). (J, K) Relative expression of PDE1A and PRKACA mRNA in the Sham, SCI, and SCI + iTBS groups by qRT‐PCR (*n* = 4 per group). (L) cAMP expression level detected by ELISA (*n* = 4 per group). The data are presented as means ± SD. Compared to the Sham group, *p < 0.05, **p < 0.01, ***p < 0.001; compared to the SCI group, ^#^ *p* < 0.05, ^##^ *p* < 0.01, ^###^ *p* < 0.001; ns *p* > 0.05 (one‐way ANOVA followed by Tukey's post hoc test [H–L]).

To further explore how dual‐target iTBS affects neurological function after SCI, we conducted DIA quantitative proteomics and metabolomics sequencing of spinal tissue from each group. We identified proteins commonly involved across the groups and obtained an intersection of 14 significantly different proteins, which include PDE1A (Figure 6E,F). KEGG enrichment analysis showed that the differentially expressed genes were mainly enriched in pathways related to synapses (Figure 6C,D). Additionally, through protein interaction network analysis, we found that PDE1A is involved in the cAMP signaling pathway. PKA is considered the primary target of cAMP, and protein kinase cAMP‐activated catalytic subunit alpha (PRKACA) is a catalytic subunit of PKA [37].

Sequencing results showed that compared to the Sham group, the expression of PDE1A in the SCI group was significantly increased, and dual‐target iTBS significantly decreased the expression of PDE1A when compared to the SCI group (Figure 6A,B). PRKACA is a catalytic subunit of PKA. Consequently, we conducted an assessment of the expression of PRKACA. The qRT‐PCR and Western blots further confirmed that dual‐target iTBS significantly decreased the expression of PDE1A and increased the expression of PRKACA after SCI (Figure 6G–K). Compared to the Sham group, the expression of cAMP in the SCI group was significantly decreased, and dual‐target iTBS significantly increased the expression of cAMP (Figure 6L). These results suggest that dual‐target iTBS may improve neurological function after SCI through the PDE1A/cAMP/PKA signaling pathway.

### 3.6 Knockdown of PDE1A Promotes Neural Growth and Synaptic Function in Primary Neurons

**FIGURE 7:**
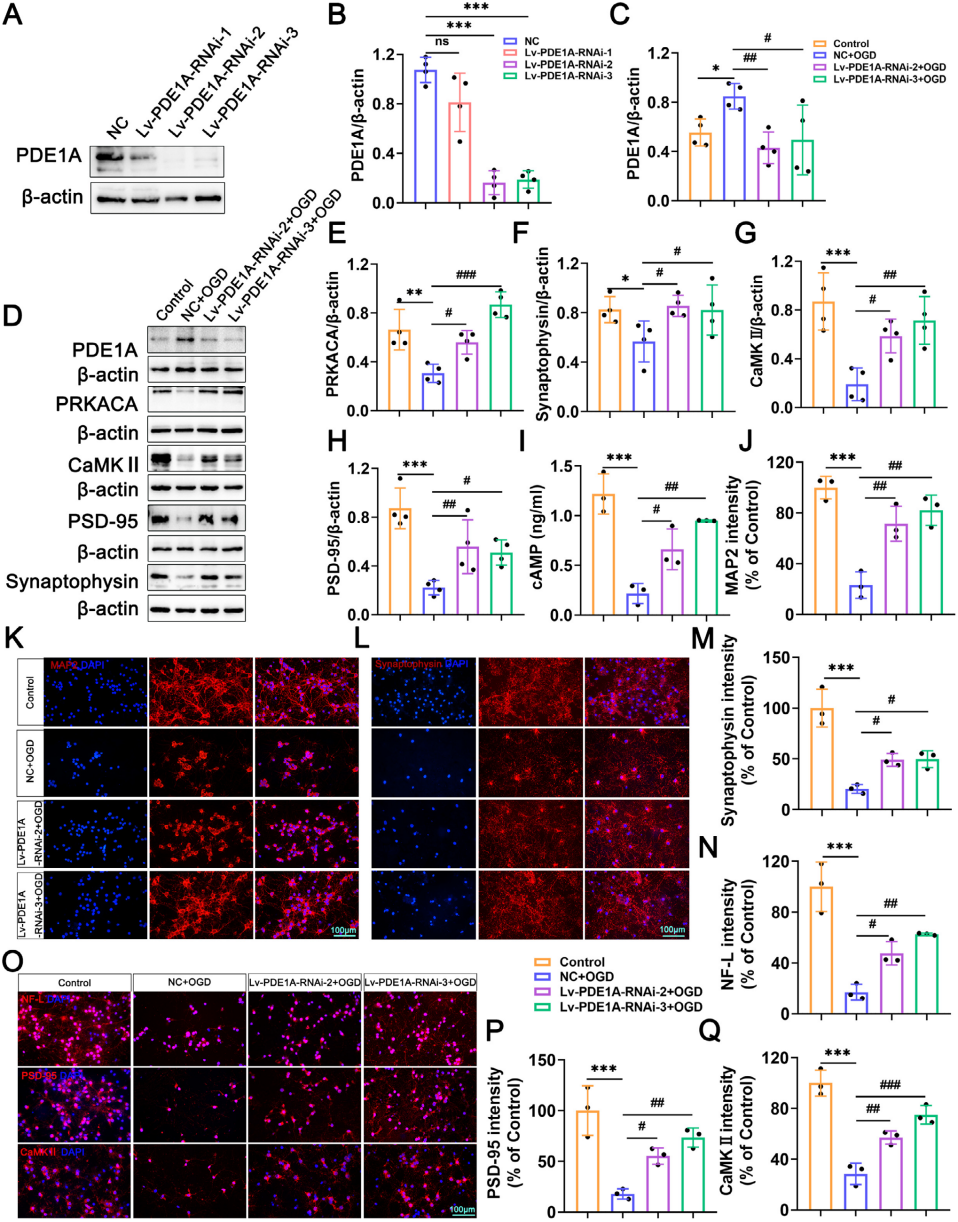
Knockdown of PDE1A promotes neural growth and synaptic function in primary neurons. (A) Western blots of PDE1A protein in the NC, Lv‐PDE1A‐RNAi‐1, Lv‐PDE1A‐RNAi‐2, and Lv‐PDE1A‐RNAi‐3 groups. (B) Quantification of PDE1A protein expression levels in A (*n* = 4 per group). (C) Quantification of PDE1A protein expression levels in D (*n* = 4 per group). (D) Western blots of PDE1A, PRKACA, Synaptophysin, PSD‐95, CaMKII proteins in the Control, NC + OGD, Lv‐PDE1A‐RNAi‐2 + OGD, and Lv‐PDE1A‐RNAi‐3 + OGD groups. (E–H) Quantification of PRKACA, Synaptophysin, CaMKII, PSD‐95 proteins expression levels in D (*n* = 4 per group). (I) cAMP expression level detected by ELISA (*n* = 3 per group). (J) Quantification of IF staining of MAP2 (*n* = 3 per group). (K, L) Representative images of IF staining of MAP2 and Synaptophysin in primary neurons. (M, N) Quantification of IF staining of Synaptophysin and NF‐L (*n* = 3 per group). (O) Representative images of IF staining of NF‐L, PSD‐95, and CaMKII in primary neurons. (P, Q) Quantification of IF staining of PSD‐95 and CaMKII (*n* = 3 per group). The data are presented as means ± SD. *p < 0.05, **p < 0.01, ***p < 0.001; ^#^ *p* < 0.05, ^##^ *p* < 0.01, ^###^ *p* < 0.001; ns *p* > 0.05 (one‐way ANOVA followed by Tukey's post hoc test [B, C, E–G, H–J, M, N, P, Q]).

To further investigate how PDE1A affects neural regeneration and synaptic function, we transduced primary neurons with lentivirus to knock down PDE1A. The knockdown efficiency of the lentivirus was verified in normal primary neurons. The results showed that, compared to the NC group, the expression level of PDE1A protein was significantly reduced in the Lv‐PDE1A‐RNAi‐2 and Lv‐PDE1A‐RNAi‐3 groups. However, there was no significant difference in the expression of PDE1A protein between the Lv‐PDE1A‐RNAi‐1 group and the NC group (Figure 7A,B). Therefore, we selected Lv‐PDE1A‐RNAi‐2 and Lv‐PDE1A‐RNAi‐3 lentivirus for subsequent experiments.

On the third day of primary neuron culture, negative control lentivirus and PDE1A knockdown lentivirus were transfected. Three days post‐transduction, the OGD model was established. As shown in Figure 7C–I, PDE1A knockdown upregulated PRKACA and cAMP and increased the expression of synaptic proteins such as Synaptophysin, PSD‐95, and CaMKII. The results of IF staining demonstrated that inhibiting PDE1A promoted neurite outgrowth and synapse formation (Figure 7J–Q). These results indicate that inhibiting PDE1A activates the cAMP/PKA signaling pathway and promotes neurite outgrowth and synaptic function in primary neurons.

### 3.7 Knockdown of PDE1A Improves Neurological Function After SCI

**FIGURE 8:**
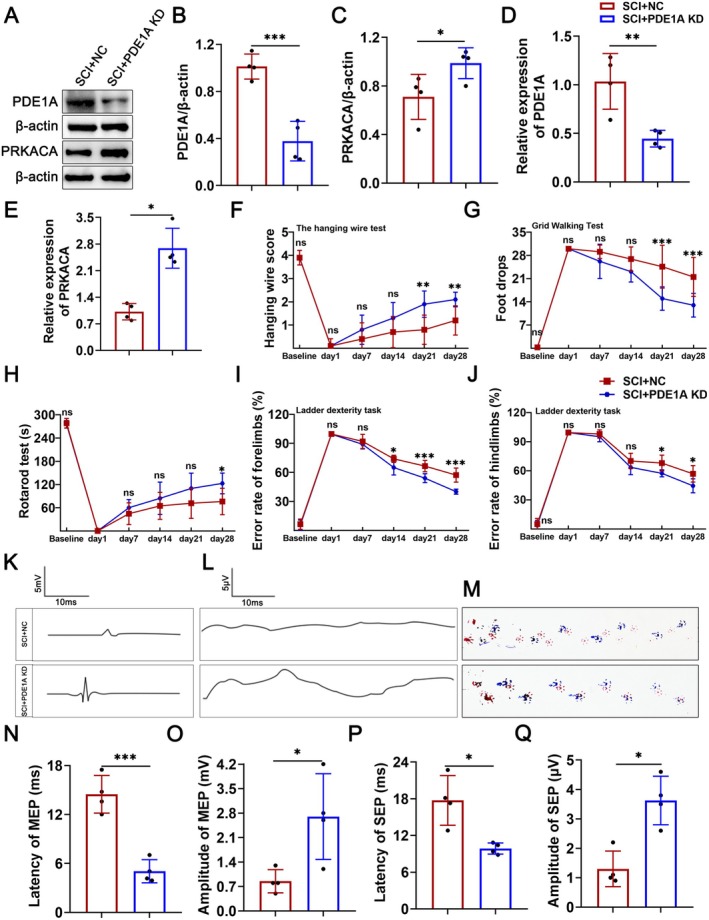
Knockdown of PDE1A improves neurological function after SCI. (A) Western blots of PDE1A, PRKACA proteins in the SCI + NC, SCI + PDE1A KD groups. (B, C) Quantification of PDE1A and PRKACA proteins expression levels in A (*n* = 4 per group). (D, E) Relative expression of PDE1A and PRKACA mRNA in the SCI + NC, SCI + PDE1A KD groups by qRT‐PCR (*n* = 4 per group). (F) The hanging wire test over time (*n* = 10 per group). (G) The grid walking test over time (*n* = 10 per group). (H) The rotarod test over time (*n* = 10 per group). (I) The error rates of forelimbs in the ladder dexterity test over time (*n* = 10 per group). (J) The error rates of hindlimbs in the ladder dexterity test over time (*n* = 10 per group). (K) Representative images of MEP. (L) Representative images of SEP. (M) Representative images of footprint (*n* = 4 per group). (N) Quantification of MEP and latency period in K (*n* = 4 per group). (O) Quantification of MEP and amplitude period in K (*n* = 4 per group). (P) Quantification of SEP and latency period in L (*n* = 4 per group). (Q) Quantification of SEP and amplitude period in L (*n* = 4 per group). The data are presented as means ± SD. ns *p* > 0.05, *p < 0.05, **p < 0.01, ***p < 0.001 (unpaired *t*‐test [B–E, N, O, Q], and Welch's *t*‐test [P]; two‐way ANOVA followed by Bonferroni post hoc test [F–J]).

To verify the specific effects of PDE1A knockdown after SCI, we transfected lentivirus around the injured spinal cord segments of SCI rats, and then assessed tissue repair and neural function of the rats. As shown in Figure 8A–E, inhibiting PDE1A significantly increased the expression of PRKACA in spinal cord tissue after SCI.

Subsequently, we assessed the motor function of the rats. The results of the ladder dexterity test revealed that 14, 21, and 28 days post‐lentivirus transfection, the error rates of forelimbs in the SCI + PDE1A KD group were significantly lower when compared with the SCI + NC group (Figure 8I). At 21 and 28 days post‐transfection, PDE1A knockdown was associated with significantly higher scores in the hanging wire test, as well as a reduction in the number of foot slips in the grid walking test and the error rates of hindlimbs following SCI (Figure 8F,G,J). Additionally, there were longer drop times observed in the rotarod test after 28 days of PDE1A knockdown (Figure 8H). Furthermore, footprint analysis indicated a significant improvement in gait coordination following PDE1A knockdown after SCI (Figure 8M). These results suggest that inhibiting PDE1A enhances motor function in rats with SCI.

To assess the neural conduction function of the rats, we conducted MEP and SEP tests (Figure 8K,L). The results showed that knockdown of PDE1A increased the amplitudes of MEP and SEP while reducing their latencies, indicating an improvement in neural conduction function in SCI rats (Figure 8N–Q). These results suggest that inhibiting PDE1A enhances neurological function in SCI rats.

### 3.8 Knockdown of PDE1A Improves Neural Regeneration After SCI

**FIGURE 9:**
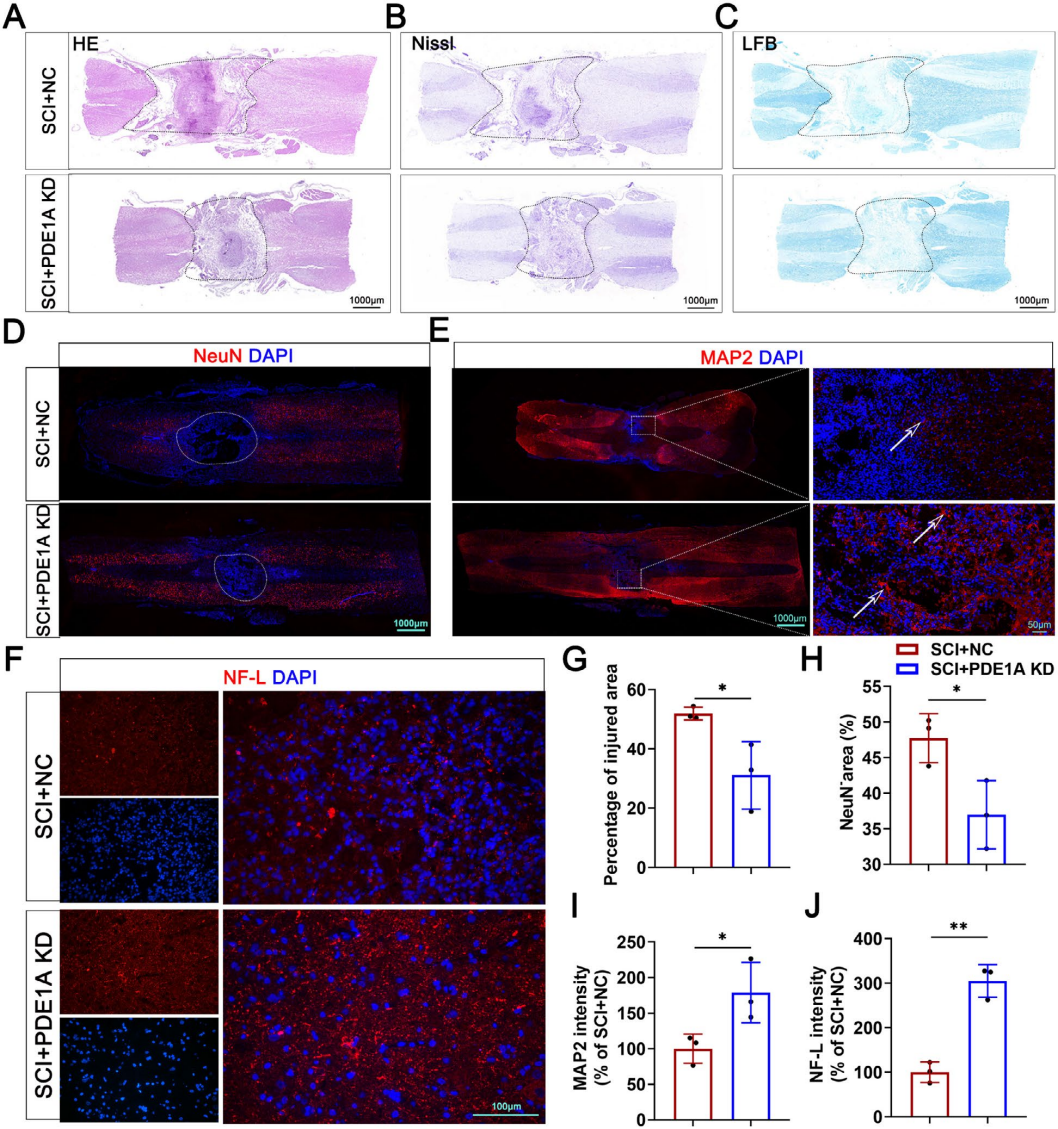
Knockdown of PDE1A improves neural regeneration after SCI. (A) Representative images of H&E staining. (B) Representative images of Nissl staining. (C) Representative images of LFB staining. (D) Representative images of IF staining of NeuN. (E) Representative images of IF staining of MAP2. (F) Representative images of IF staining of NF‐L. (G) Quantification of H&E staining in A (*n* = 3 per group). (H) Quantification of IF staining of NeuN‐negative area in D (*n* = 3 per group). (I) Quantification of IF staining of MAP2 in E (*n* = 3 per group). (J) Quantification of IF staining of NF‐L in F (*n* = 3 per group). The data are presented as means ± SD. ns *p* > 0.05, *p < 0.05, **p < 0.01, ***p < 0.001 (unpaired *t*‐test [G–J]).

We investigated the effect of PDE1A knockdown on the structural repair of injured spinal cord tissue. The results indicated that PDE1A knockdown significantly reduced the lesion area associated with SCI (Figure 9A–C,G). Additionally, the expression of NeuN, MAP2, and NF‐L in spinal cord tissue was assessed using IF staining. As demonstrated in Figure 9D–F,H–J, PDE1A knockdown significantly increased the expression of MAP2 in spinal cord lesions following SCI, reduced the NeuN‐negative area, and increased the expression of NF‐L. These findings suggest that inhibiting PDE1A enhances neural regeneration after SCI.

### 3.9 Knockdown of PDE1A Improves Synaptic Remodeling After SCI

**FIGURE 10:**
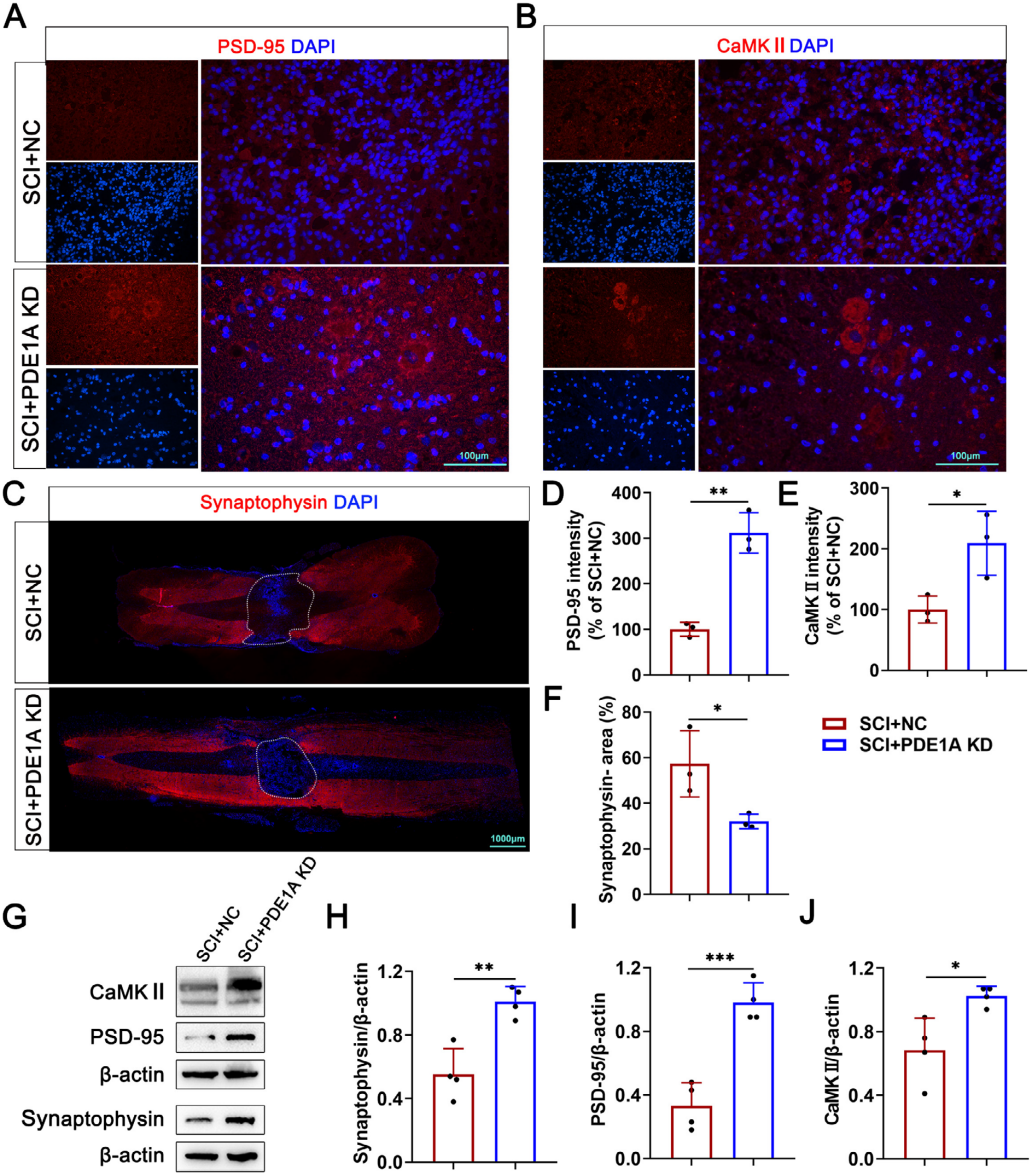
Knockdown of PDE1A improves synaptic remodeling after SCI. (A–C) Representative images of IF staining of PSD‐95, CaMKII, and Synaptophysin. (D–F) Quantification of IF staining of PSD‐95, CaMKII, and Synaptophysin‐negative area in A–C (*n* = 3 per group). (G) Western blots of Synaptophysin, PSD‐95, and CaMKII proteins in the SCI + NC and SCI + PDE1A KD groups. (H–J) Quantification of Synaptophysin, PSD‐95, and CaMKII proteins expression levels in G (*n* = 4 per group). The data are presented as means ± SD. ns *p* > 0.05, *p < 0.05, **p < 0.01, ***p < 0.001 (unpaired *t*‐test [D–F, H–J]).

To evaluate the effect of PDE1A knockdown on synaptic function following SCI, we assessed the expression of Synaptophysin, PSD‐95, and CaMKII using IF staining and Western blot assays. The results demonstrated that PDE1A knockdown significantly increased the expression of Synaptophysin, PSD‐95, and CaMKII and significantly reduced the Synaptophysin‐negative area in spinal cord tissue following SCI (Figure 10A–J). These findings indicate that the inhibition of PDE1A promotes synaptic remodeling in neurons following SCI.

## 4 Discussion

There are limited effective treatment methods after SCI, necessitating the development of new therapeutic strategies. In this study, we found that transcranial iTBS combined with trans‐spinal iTBS improved neurological function in SCI rats and promoted neural regeneration and synaptic remodeling. Further investigations revealed that PDE1A was upregulated, while PRKACA and cAMP were downregulated following SCI. The inhibition of PDE1A led to the upregulation of cAMP levels in primary neurons and promoted neural growth and the expression of synaptic‐related proteins. Additionally, inhibiting PDE1A improved neurological function in SCI rats and effectively promoted synaptic remodeling. Our results confirmed the efficacy of dual‐target iTBS for neural regeneration after SCI, potentially regulated through the PDE1A‐cAMP‐PKA signaling pathway.

As a noninvasive neuromodulation technique, TMS shows promise in the treatment of various neurological conditions. Recent research indicates that applying single magnetic stimulation to either the cortex or spinal cord is beneficial for axonal regeneration and neural function [38, 39]. For instance, Hu et al. [40] found that high‐frequency repetitive TMS (rTMS) on the cerebral cortex of SCI rats promoted the restoration of the integrity of the corticospinal tract and the regeneration of spinal cord axons, with a significant increase in the expression of GAP‐43 and SYN1 in the spinal cord. Furthermore, repeated trans‐spinal magnetic stimulation (rTSMS) enhanced the phagocytic activity of microglia to remove myelin debris, improved the microenvironment after SCI, and promoted functional recovery post‐SCI [41]. However, it remains unclear whether combined stimulation of these two targets is effective for treating neurological dysfunction following SCI. Our preliminary clinical studies have confirmed that dual‐target iTBS significantly improved bilateral lower limb motor function in patients with incomplete SCI, with noticeable increases in amplitude and significant decreases in latency of MEP, indicating that dual‐target iTBS may be involved in the reconstruction and repair of certain neural circuits following incomplete SCI [18]. In this study, we compared the effects of single‐target versus dual‐target iTBS on SCI in rats. The results showed that, compared to single‐target iTBS, dual‐target iTBS significantly improved motor function and spinal cord tissue repair in rats with SCI, and dual‐target iTBS improved synaptic remodeling and promoted neural repair. Transcranial iTBS directly stimulates the sensorimotor cortex, activating neurons in the brain and modulating the immune microenvironment to achieve neuroprotective effects and initiate brain‐derived axonal regeneration [34]. Moreover, direct magnetic stimulation at the segment of SCI modulated scar formation, supported neuronal survival, and promoted axonal regeneration [42]. Stimulation of the cerebral cortex effectively activates the descending corticospinal tract, whereas stimulation of the spinal cord and nerve roots can activate ascending sensory pathways. This approach may be effective in promoting neural circuit remodeling.

To further investigate the mechanisms through which dual‐target iTBS enhances neurological function following SCI, we performed metabolomic and proteomic sequencing analyses on spinal cord tissue samples from each experimental group. The results revealed a significant increase in PDE1A expression after SCI, while the dual‐target iTBS intervention resulted in a reduction of PDE1A expression. Additionally, we observed that iTBS increased the expression of cAMP and PRKACA in rats with SCI. As a member of the PDE family, PDE1A hydrolyzes cAMP or cGMP, thereby inhibiting cyclic nucleotide signaling [43, 44]. The interaction between cAMP and the regulatory subunits of PKA induces a conformational change that permits the release of the catalytic subunits, which subsequently phosphorylate downstream targets to mediate various cellular functions [45]. PRKACA serves as a catalytic subunit of PKA, and the regulatory and catalytic subunits of PKA reorganize to return to their inactive state following cAMP hydrolysis by PDE [46]. The expression of cAMP decreases after SCI, creating an unfavorable environment for neural regeneration [47]. Prior research has suggested that elevated cAMP levels promote axonal growth and neuronal survival across multiple species [48, 49]. Thus, increasing cAMP levels emerges as a viable strategy to enhance neural regeneration following SCI [27]. Research has reported that PDE4 inhibitors enhanced axonal regeneration and functional recovery following SCI [50, 51]. Specific PDE inhibitors increased cAMP levels in the spinal cord and medulla oblongata after high cervical SCI while enhancing diaphragm motor output and improving respiratory function [52]. In our study, iTBS inhibited the expression of PDE1A in spinal cord tissue after SCI and significantly upregulated the expression of PRKACA, promoting synaptic remodeling. Additionally, inhibiting PDE1A increased the expression of cAMP and PRKACA in primary neurons, promoted the expression of synaptic‐related proteins, suggesting that the cAMP/PKA signaling pathway may be activated, thereby enhancing synaptic plasticity. Therefore, PDE1A may be a potential molecular target for regulating synaptic function after SCI.

Nonetheless, there are still limitations in this study. First, the pathological changes after traumatic SCI are extremely complex, and these changes may not be fully replicated through the in vitro OGD/R model of primary neurons. Moreover, the precise mechanisms by which the cAMP/PKA signaling pathway regulates neural regeneration following SCI remain inadequately understood, presenting an important area for further investigation by our research team.

In summary, this study illustrates that transcranial iTBS combined with trans‐spinal iTBS improves neural regeneration and synaptic reconstruction after SCI, possibly by activating the PDE1A‐cAMP‐PKA signaling pathway. This research explores new targets for magnetic stimulation in enhancing neural regeneration after SCI and provides a theoretical foundation for clinical applications of magnetic stimulation in promoting functional recovery in SCI.

## Author Contributions

**Yingxue Fu:** writing – original draft, visualization, validation, investigation, formal analysis, conceptualization. **Xianbin Wang:** writing – review and editing, supervision, resources, methodology, funding acquisition. **Xingyu Chen:** investigation, formal analysis. **Shuang Wu:** project administration, writing – review and editing, supervision, funding acquisition.

## Conflicts of Interest

The authors declare no conflicts of interest.

## Data Availability

The data that support the findings of this study are available on request from the corresponding author. The data are not publicly available due to privacy or ethical restrictions.
